# Unveiling the invisible: mathematical methods for restoring and interpreting illuminated manuscripts

**DOI:** 10.1186/s40494-018-0216-z

**Published:** 2018-09-24

**Authors:** Luca Calatroni, Marie d’Autume, Rob Hocking, Stella Panayotova, Simone Parisotto, Paola Ricciardi, Carola-Bibiane Schönlieb

**Affiliations:** 1 CMAP, École Polytechnique, Route de Saclay, 91128 Palaiseau, France; 2 CMLA, ENS Cachan, CNRS, Université Paris-Saclay, 61 Avenue President Wilson, 94235 Cachan, France; 3 DAMTP, University of Cambridge, Wilberforce Road, Cambridge, CB3 0WA UK; 4 CCA, University of Cambridge, Wilberforce Road, Cambridge, CB3 0WA UK; 5 Fitzwilliam Museum, University of Cambridge, Trumpington Street, Cambridge, CB2 1RB UK

**Keywords:** Sample, Mathematical image reconstruction, Image inpainting, Image osmosis, 3D visualisation

## Abstract

The last 50 years have seen an impressive development of mathematical methods for the analysis and processing of digital images, mostly in the context of photography, biomedical imaging and various forms of engineering. The arts have been mostly overlooked in this process, apart from a few exceptional works in the last 10 years. With the rapid emergence of digitisation in the arts, however, the arts domain is becoming increasingly receptive to digital image processing methods and the importance of paying attention to this therefore increases. In this paper we discuss a range of mathematical methods for digital image restoration and digital visualisation for illuminated manuscripts. The latter provide an interesting opportunity for digital manipulation because they traditionally remain physically untouched. At the same time they also serve as an example for the possibilities mathematics and digital restoration offer as a generic and objective toolkit for the arts.

**Electronic supplementary material:**

The online version of this article (10.1186/s40494-018-0216-z) contains supplementary material, which is available to authorized users.

## Introduction

The digital processing, analysis and archiving of databases and collections in the arts and humanities is becoming increasingly important. This is because of a myriad of possibilities that digitisation opens up that go well beyond the organisation and manipulation of the actual physical objects, allowing, for instance, the creation of digital databases that are searchable with respect to several parameters (keywords), the digital processing and analysis of objects that are non-destructive to the original object, and the application of automated algorithms for sorting newly found objects into existing digital databases by classifying them into pre-defined groups in the database. These possibilities go hand-in-hand with ever-growing advances in data science that are developing mathematical methodology for analysing and processing digital data. A large component of digital data in the arts and humanities is composed of digital images. Despite many developments of mathematical image analysis methods in applications like biomedicine, the physical sciences and various forms of engineering, the arts and humanities have been mostly overlooked as an application in need of bespoke mathematical image analysis methods. Still, a few examples in this context exist and encompass works on forgery detection [1], the digital restoration of paintings with the Ghent Altarpiece [2–7] and Van Gogh’s Field with Irises [8–10] being prominent examples in these efforts, the digitally guided restoration of frescoes as done for the Mantegna frescoes [11, 12] and the Neidhart frescoes [13, 14], the algorithm-based analysis and classification of texture in paintings [15, 16], learned representations of artists’ styles and painting techniques [17, 18], and multi-modal image registration and colour analysis in paintings [19–23], just to name a few.

In this work we discuss a range of mathematical methods for correcting and enhancing images of illuminated manuscripts. In particular, we consider automated and semi-automated models for digital image restoration based on partial differential equations, exemplar-based image inpainting and osmosis filtering, and their translation to the digital interpretation of illuminated manuscripts. Here, we refer to mathematical image processing as the task of digital image restoration (or reconstruction), that is the digital processing of a given image to correct for its visual imperfections. Generally, this is done with the main intention of producing a final result where imperfections have been corrected in a visually least distracting way. This is the case for several imaging tasks such as image denoising, deblurring and also image inpainting.

Medieval and Renaissance illuminated manuscripts present a particular challenge, but also an opportunity to transform current understanding of European visual culture between the 6th and 16th century. Illuminated manuscripts are the largest and best preserved resource for the study of European painting before 1500. Nevertheless, the images in some manuscripts have been affected by wear-and-tear, degradation over time, iconoclasm, censorship or updating. Unlike the conservation of other painted artefacts, the conservation of illuminated manuscripts preserved in institutional collections is non-invasive, usually restricted to repairs of the binding and of torn parchment or paper, and rarely involves the consolidation of flaking pigments. For the study of illuminated manuscripts, physical restoration and repairs are often disregarded. This minimal approach is due largely to the fact that when compared to wall or easel paintings, the images in illuminated manuscripts are relatively small and their pigment layers are few and very delicate. It is not possible to remove over-painting without damaging or completely removing the original painting beneath. The removal of even the smallest sample or the restoration of even the smallest painted area would constitute a considerable change to the overall image. As a consequence, pigment losses are often not filled in and over-paintings added on top of the superficial layers can often not be removed to reveal the original images. Virtual restoration is thus the only way to recover damaged illuminations, whether by infilling paint losses or by removing over-painted layers or indeed both. Bringing the images as close as possible to their original form would ensure both their accurate scholarly interpretation and their full appreciation by wider audiences. Damaged or inaccurately restored illuminations can lead to the exclusion of seminal works of art from academic debates or to incomplete and misleading interpretations of the dating, origin and artists involved. Preserving the current state of the illuminations in line with conservation ethics, faithful digital restoration would serve as a reliable surrogate for multiple reconstructions, enabling research, teaching and wider appreciation for manuscripts.

The reliable processing of illuminated manuscripts requires a multi-disciplinary collaboration as the current work is based on. In what follows we discuss a range of new adaptive, semi-automated restoration methods that (a) reconstruct image-structures using partial differential equations [13, 14, 24–28], (b) mimic the human-expert behaviour, using texture- and structure patches sampled from the intact part of the illuminated manuscript at hand and integrating them in exemplar-based inpainting approaches [29, 30] in order to provide a digital restoration in agreement with the available information and pleasant to the eye (c) exploit infrared imaging data, correlating the visible image content with its traces in the hidden layers of paint [31, 32], and (d) create new 3D interpretations of illuminated manuscripts through a new 3D conversion pipeline [33]. The pre-sequel of this work is an article in the exhibition catalogue [32].

*Organisation.* In “Retrieving missing contents via image inpainting” section we propose a semi-supervised approach for the segmentation of damaged areas of colour accurate images (in the following referred to simply as RGB images) of illuminated manuscripts and for the retrieval of missing information via a two-step image inpainting model. In “Looking through the layers via osmosis filtering” section we consider the mathematical model of image osmosis to integrate super-painted visible image information on a manuscript with hidden infrared ones for looking through the layers of a restoration process. Finally, in “Creating a 3D virtual scene from illuminated manuscripts” section we present a mathematical pipeline to convert a 2D painting into a 3D scene by means of the construction of an appropriate depth map.

## Retrieving missing contents via image inpainting

The problem of image inpainting can be described as the task of filling in damaged (or occluded) areas in an image *f* defined on a rectangular domain $\Omega$ by transferring the information available in the intact areas of the image to the damaged areas in the image. Over the last 30 years a large variety of mathematical models solving the image inpainting problem have been proposed, see, e.g., [28, 34] for a review. In some of them, image information is transferred into the damaged areas (the so-called *inpainting domain*, denoted by *D* in the following) by using *local* information only, i.e. by means of suitable diffusion and transport processes which interpolate image structures in the immediate vicinity of the boundary of *D* in the occluded region. Such techniques have been shown to be effective for the transfer of geometric image structures, even in the presence of large damaged areas [28]. However, because of their local nature, such methods do not make use of the entire information contained in the intact image regions. In particular, such methods do not take into account non-local image information in terms of patterns and textures nor image contents located far away of *D*. For this reason, non-local mathematical models exploiting self-similarities in the whole image have been proposed [29, 30, 35, 36]. Such models operate on image patches rather than single pixels. Small patches inside *D* are iteratively reconstructed by comparison with patches outside *D* in a suitable distance. Missing patches are then reconstructed by copy and paste of a closest patch (or its centre pixel) from the intact part of the image. These models have been proven to be impressively effective in a very large variety of applications and rendered computationally feasible in recent years with the well-known PatchMatch algorithm [37].

The first step of any inpainting algorithm is the decomposition of the image domain in damaged and undamaged areas. This is an image segmentation problem, decomposing a given image into its constituting regions, cf. for instance [34]. Its solution may be rendered very hard in the presence of fuzzy and irregular region boundaries and small scale objects.

**Fig. 1:**
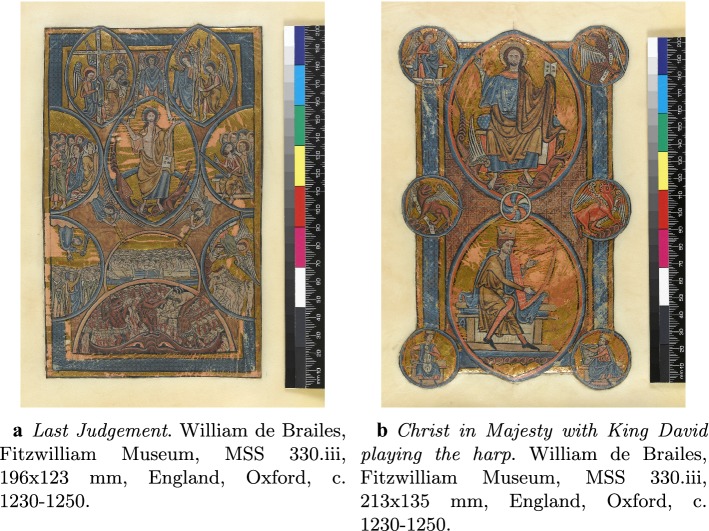
Illuminated manuscripts. These two illuminated manuscripts show large and non-homogeneous damaged areas, mainly removal of gold leaves, see “Retrieving missing contents via image inpainting” section for more details

In the following we describe an algorithm which detects damaged areas in images with possibly large and non-homogeneous missing regions using few examples provided by the user. This is then used as a necessary initial step for the subsequent application of a two-stages inpainting procedure based on total variation inpainting [38] and exemplar-based image inpainting proposed in [36] for the reconstruction of image contents in the images of the illuminated manuscripts in Fig. 1. Our proposed segmentation is *semi-supervised* since user input is required for training, while the inpainting procedure is fully automated.

### Description of the dataset

Our dataset is composed of two manuscripts made by William de Brailes in 1230-1250 and now part of the collection of the Fitzwilliam Museum in Cambridge (UK), see Fig. 1: *Last Judgement* in Fig. 1a and *Christ in Majesty with King David playing the harp* in Fig. 1b, of dimension 196x123mm and 213x135mm, respectively. The images are acquired with a Leaf Valeo 22 back utilising a Mamyia RB67 body and the resulting RAW files are processed using Leafs own proprietary software, where distortions and aberrations are corrected. Also, the colour accuracy is provided by using a customized Kodak colour separation guide with grey-scale (Q13 equivalent) and exported in Adobe 98 colour space. The final output results in very large .tif images (about $4008\times 5344$ pixels and 47 MB each).

### A semi-supervised algorithm for the detection of the damaged areas

For identifying the damaged areas in the image (mainly missing gold leaves) we propose in the following a two-step semi-supervised algorithm. Here, a classical binary segmentation model is used first for the extraction of a small training region as described in “Chan-Vese segmentation” section which subsequently serves as an input for a labelling algorithm which segments the whole inpainting domain based on appropriate intensity-based image features in “Image descriptors: feature extraction” and “A clustering algorithm with training” sections.

#### Chan-Vese segmentation

In binary image segmentation one seeks to partition an image in two disjoint regions, each characterised by distinctive features. Typically, RGB intensity values are used to describe image contents and mathematical image segmentation methods often compute the required segmented image as the minimiser of an appropriate functional.

Let *f* be the given image. We seek a binary image *u* so that

1 $$\begin{aligned} u(x) = {\left\{ \begin{array}{ll} c_1, & {}\quad {\text {if}} \; x \text{ is } \text{ inside } C, \\ c_2, & {} \quad {\text {if}} \; x \text{ is } \text{ outside } C, \end{array}\right. } \end{aligned}$$

where *C* is a closed curve. In this work, we consider the Chan-Vese segmentation functional for binary image segmentation [39], that is

2 $$\begin{aligned} \mathcal {F}(c_1,c_2,C):= \, & {} \mu ~ \text {Length}(C) + \nu ~\text {Area}\left( int(C)\right) \\&+ \nonumber \lambda _1\sum _{x\in int(C)} | f(x)-c_1 |^2 + \lambda _2\sum _{x\in ext(C)} |f(x)-c_2|^2. \end{aligned}$$

The functional $\mathcal F$ is minimised for constants $c_1$ and $c_2$ and the contour *C*, i.e. the optimal *u* of the form (1). Here, $\mu ,\,\nu ,\,\lambda _1,\,\lambda _2>0$ are positive parameters and $int(C),\, ext(C)$ denote the inner and the outer part of *C*, respectively. In (2) the first and second term penalise the length of *C* and the area of the region inside *C*, respectively, giving control on the smoothness of *C* and the size of the regions. The two other terms penalise the discrepancy between the fitting of the piecewise constant *u* in (1) and the given image *f* in the interior and exterior of *C*, respectively. By computing a minimum of (2) one retrieves a binary approximation *u* of *f*.

Despite being very popular and widely used in applications, the Chan-Vese model and its extensions present intrinsic limitations. Firstly, the segmentation result is strongly dependent on the initialisation: in order to get a good result, the initial condition needs to be chosen within (or sufficiently close to) the domain one aims to segment. Secondly, due to the modelling assumption (1), the Chan-Vese model works well for images whose intensity is locally homogeneous. If this is not the case, the contour curve *C* may evolve along image information different from the one we want to detect. Images with significant presence of texture, for instance, can exhibit such problems. Furthermore, the model is very sensitive to the length and area parameters $\mu$ and $\nu$, which may make the segmentation of very small objects in the image difficult.

**Fig. 2:**
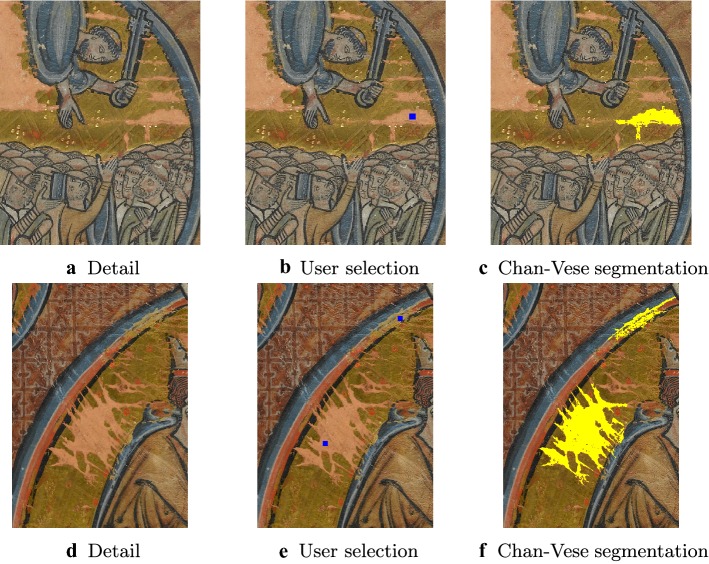
First step in the detection of the damaged regions. This detection is based on the Chan-Vese segmentation described in “Chan-Vese segmentation” section. The user clicks on the damaged region to select training pixels (in blue) which serve as initialisation of the Chan-Vese model (1). The segmentation algorithm is run and the training region $D_1$ inside the damaged area is segmented. The result is superimposed on the given image and coloured in yellow for better visualisation

For our application, we make use of the Chan-Vese model^1^ to segment a sub-region $D_1$ of *D* that will serve as a training set for the classification described in the following two subsections. To do that, we ask the user (typically, an expert in the field) simply to click on a few pixels inside the inpainting domain *D* to identity a candidate initial condition for the segmentation model (1), which is then run to segment the subregion $D_1$. In Fig. 2 we show the results of this approach with a superimposed mask of the computed region $D_1$ for some details cropped from the original images.

Because of the intrinsic limitations of the Chan-Vese approach, we observe that the segmentation result is not satisfactory (see, for instance, the example in the first row of Fig. 2) since it generally detects with high precision only the largest uniform region around the user selection. To detect the whole inpainting domain *D* in this manner, the user should in principle give many initialisation points, which may be very demanding in the presence of several disconnected and possibly tiny inpainting regions.

For this reason, we proceed differently and make use of a feature-based approach to use the area $D_1$ as a training region for a clustering algorithm running over the whole set of image pixels. This procedure is described in the next two sections.

#### Image descriptors: feature extraction

In order to describe the different regions in the image in a distinctive way, we consider intensity-type features. Namely, for every pixel *x* in the image we apply non-linear colour transformations to compute the HSV (Hue, Saturation, Value), the geometric mean chromaticity GMCR [40], the CIELAB and the CMYK (Cyan, Magenta, Yellow, Key) values (see [41] for more details). Once this is done, we append all these values and store them in a feature vector $\varvec{\psi }$ of the form

3 $$\begin{aligned} \varvec{\psi }(x)= [ \text {HSV}(x), \text {GMCR}, \text {CIELAB}(x), \text {CMYK}(x)]. \end{aligned}$$

For our purpose the feature vector (3), essentially based on RGB intensities, rendered precise segmentations. For more general segmentation purposes, one could add texture-based features and, if available, multi-spectral measurements such as infrared IR or ultraviolet UV images.

#### A clustering algorithm with training

**Fig. 3:**
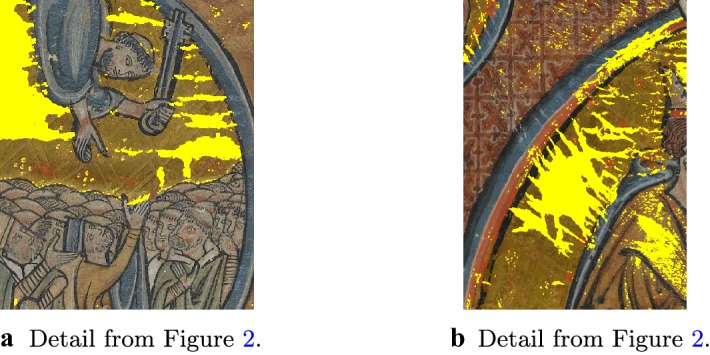
Second step in the detection of the damaged region. The *k*-means clustering algorithm is run on the whole image selection in terms of intensity-based image features, cf. “Image descriptors: feature extraction” and “A clustering algorithm with training” sections. The outputs of the binary segmentation algorithm shown in Fig. 2 are used as guidance for the clustering algorithm

Once the feature vectors are built for every pixel in the image, we use the training region $D_1$ detected as described in “Chan-Vese segmentation” section as a dictionary to drive the segmentation procedure extended to the whole image domain. We proceed as follows. First, we run a clustering algorithm over the whole image domain comparing the features defined in (3) in order to partition the image in a fixed number of *K* clusters. To do that, we use the well-known *k*-means algorithm.^2^ After this preliminary step, we check which cluster has been assigned to the training region $D_1$ and simply identify in the clustered image which pixels lie in the same cluster. By construction, this corresponds to finding the regions in the image ‘best-fitting’ the training region in terms of the features defined in “Image descriptors: feature extraction” section, which is our objective. After a refinement step based on erosion/dilation of extracted regions, so as to remove or fill-in possibly misclassified pixels, we can finally extract the whole area to inpaint *D*. We report the results corresponding to Fig. 2 in Fig 3a, b.

### Inpainting models

Once an accurate segmentation of the damaged areas is provided, the task becomes the actual restoration of the image contents in *D* by means of the available information in the region $\Omega \setminus D$. A standard mathematical approach solving such an inpainting problem consists in minimising an appropriate function $\mathcal {E}$ defined over the image domain $\Omega$, i.e. in

4 $$\begin{aligned} \text { finding }\qquad u\qquad \text {s.t.}\qquad u\in \text {argmin}_v~ \mathcal {E}(v). \end{aligned}$$

A standard choice for $\mathcal {E}$ in the case of *local* inpainting models is the functional

5 $$\begin{aligned} \mathcal {E}(v) = R(v) + \lambda \Vert \upchi _{\Omega \setminus D}(f- v)\Vert ^2_2, \end{aligned}$$

where *f* denotes the given image to restore, $\Vert \cdot \Vert _2$ is the Euclidean norm, $\lambda$ and appropriately chosen positive parameter and $\upchi _{\Omega \setminus D}$ denotes the characteristic function of the non-occluded image areas, so that for every pixel $x\in \Omega$:

$$\begin{aligned} \upchi _{\Omega \setminus D}(x) = {\left\{ \begin{array}{ll} 1\quad &{}\text {if }\; x\in \Omega \setminus D\\ 0 \quad &{} \text {if }\; x\in D. \end{array}\right. } \end{aligned}$$

The second term in (5) is as a distance function between the given image *f* and the sought after restored image *u* in the intact part of the image. The multiplication of $f-u$ by the characteristic function $\upchi$ implies that this term is simply zero for the points in *D*, since there is no information available, while $f-u$ for all the points in $\Omega \setminus D$ has to be as small as possible. The term *R* typically encodes local information (such as gradient magnitude) which is the responsible of the transfer of information inside *D* by means of possibly non-linear models [28, 34]. The transfer process is balanced with the trust in the data by the positive parameter $\lambda$. A classical choice of a gradient-based inpainting model consists in choosing

6 $$\begin{aligned} R(v) = \Vert \nabla v \Vert _1 = \sum _{x\in \Omega } | \nabla v(x) | \end{aligned}$$

i.e. the Total Variation of *v* [38]. As mentioned above such an image inpainting technique is not designed to transfer texture information. Furthermore, it fails in the inpainting of large missing areas. For our purposes we use (6) as an initial ‘good’ guess with which we initialise a different approach based on a non-local inpainting procedure as described in the following section.

#### Exemplar-based inpainting

We describe here the non-local patch-based inpainting procedure studied in [30, 36] and carefully described in [42] from an implementation point of view.^3^ In the following, we define for any point $x\in \Omega$ the *patch neighbourhood* $\mathcal {N}_x$ as the set of points in $\Omega$ in a neighbourhood of *x*. Assuming that the patch neighbourhood has cardinality *n*, by *patch* around *x* we denote the 3*n*-dimensional vector $P_{x} = (u(x_1), u(x_2),\ldots ,u(x_n) )$ where the points $x_i, i=1,\ldots n$ belong to patch neighbourhood $\mathcal {N}_x$. In order to measure ‘distance’ between patches, a suitable patch measure *d* can be defined, so that $d(P_{x},P_{y})$ stands for the patch measure between the patches around the two points *x* and *y*. We define then the Nearest Neighbour (NN) of $P_{x}$ as the patch $P_y$ around some point *y* minimising *d*.

For an inpainting application the task consists then in finding for each point *x* in the inpainting domain *D* the best-matching patch $P_y$ outside *D*. Assuming that each NN patch can be characterised in terms of a shift vector $\phi$ defined for every point in $\Omega$ (i.e. assuming there exists a rigid transformation $\phi$ which shifts any patch to its NN), the problem can be formulated as the minimisation problem

7 $$\begin{aligned} \min ~ \mathcal {E}(u,\phi ) = \sum _{x\in D}~ d^2\left( P_{x},P_{x+\phi (x)}\right) . \end{aligned}$$

Heuristically, every patch in the solution of the problem above is constructed in such a way that in the damaged region *D* the patch has a correspondence (in the sense of the measure *d*) with its NN patch in the intact region $\Omega \setminus D$. Following [42], we use the following distance:

8 $$\begin{aligned} d^2\left( P_{x},P_{x+\phi (x)}\right) = \sum _{y\in \mathcal {N}_{x}} \left( u(y)- u(y+\phi (x))\right) ^2. \end{aligned}$$

From an algorithmic point of view, solving the model involves two steps: the first consists in computing (approximately) the NN patch for each point in *D*, so as to provide a complete representation of the shift map $\phi$. This can be computationally expensive for large images. In order to solve this efficiently, a PatchMatch [37] strategy can be applied. Afterwards a proper image reconstruction step is performed, where for every point in *D* the actual corresponding patch is computed. We refer the reader to [42] for full algorithmic details.

A crucial ingredient for a good performance of the exemplar-based inpainting algorithm [30, 36] is its initialisation. In particular, once the inpainting domain is known, a pre-processing step where a local inpainting model, such as the TV inpainting model (5) with (6), can be run to provide a rough, but reliable initialisation of the algorithm.^4^

**Fig. 4:**
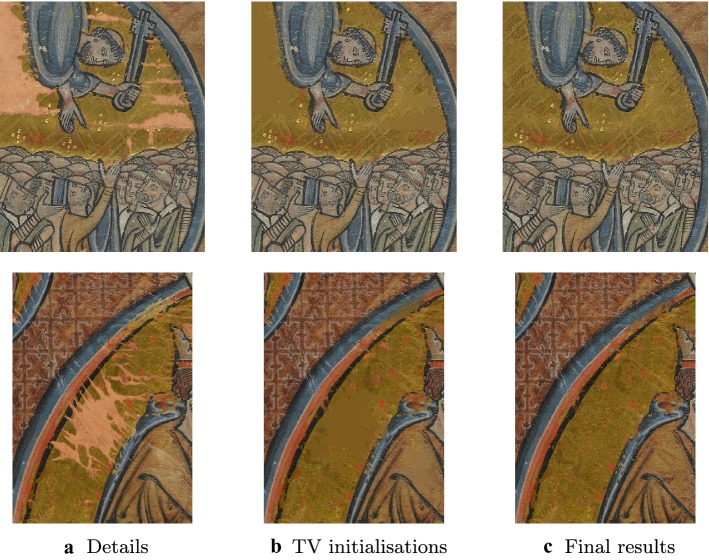
Inpainting of damaged areas in Fig. 2. Once the inpainting domain is detected, the TV inpainting model (5, 6) is used to provide a good initialisation for the exemplar-based model (7). The final result shows the desired transfer of both geometric and texture information in the damaged areas. Patch size: $5\times 5$ (upper row), $7\times 7$ (bottom row)

**Fig. 5:**
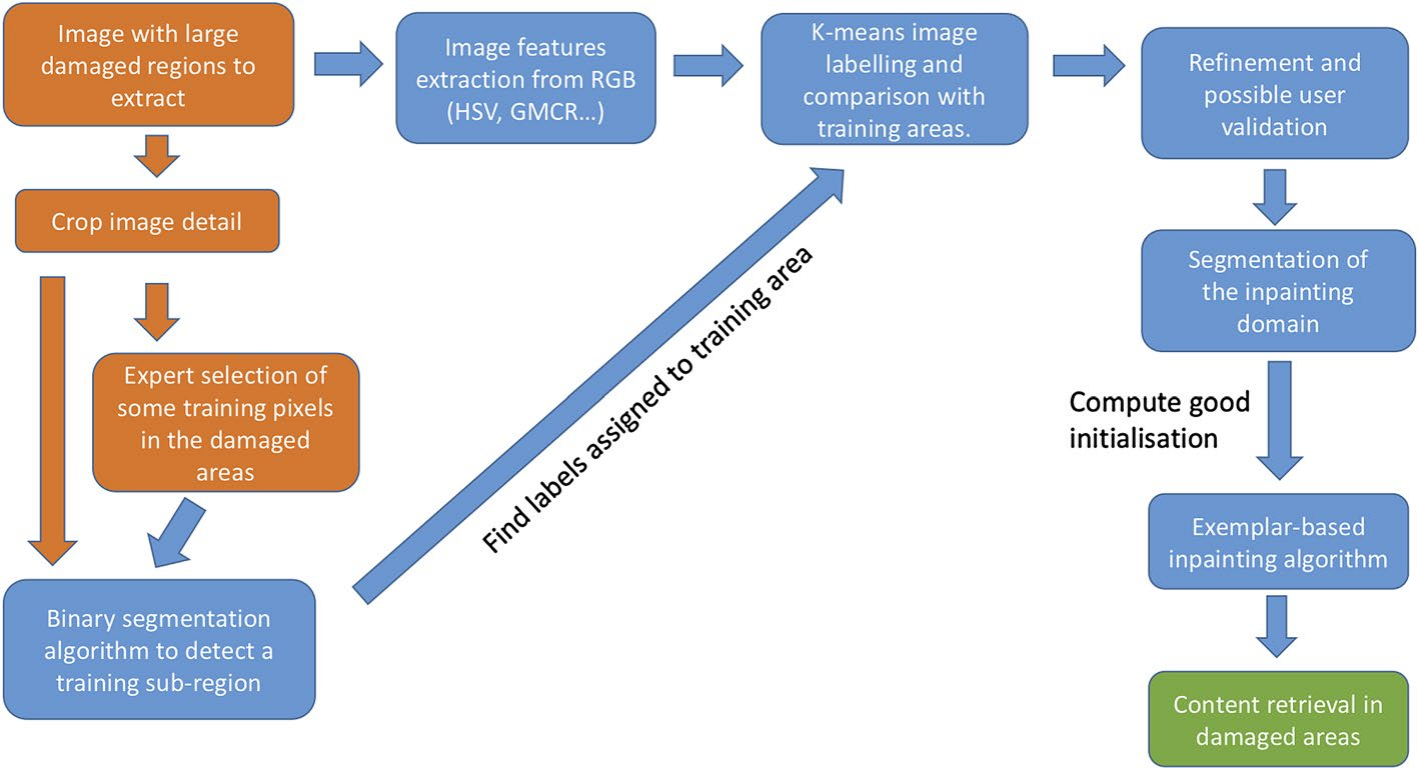
Workflow of the combined algorithm for inpainting. The diagram describes the different steps of the combined algorithm for inpainting domain detection followed by the restoration of the damaged areas via mathematical inpainting. Boxes requiring user inputs are coloured orange, whereas the ones where automatic steps are performed are coloured blue. The final objective is coloured green.

We report the results of the combined procedure in Fig. 4 and the overall work-flow of the algorithm in the diagram in Fig. 5.

#### Model parameters

For the segmentation of the training region $D_1$ within the inpainting domain *D* we use the activecontour MATLAB function by which the Chan-Vese algorithm can be called. For this we fixed the maximum number of iterations to maxiter$=1000$ and use the default value as a tolerance on the relative error between iterates as a stopping criterion. We use the default values for the parameters $\mu$ and $\nu$ in (2). The subsequent clustering phase was performed by means of the standard MATLAB kmeans function after specifying a total of $K=35$ labels to assign. The use of such a large value for *K* turned out to be crucial for an accurate discrimination. The automatic choice of the value of *K* for this type of applications is a matter of future research. The clustering was iteratively repeated 5 times to improve accuracy. Once the detection of the inpainting domain is completed, in order to provide a good initialisation to the exemplar-based model we use the TV inpainting model (4) with (6) with the value $\lambda =1000$ and a maximum number of iterations equal to maxiter2$=1000$ with a stopping criterion on the relative error between iterates depending on a default tolerance. Finally, we followed [42] for the implementation of the exemplar-based inpainting model: for this we specified 12 propagation of iterations and tested different sizes for the patches. In order to avoid memory shortage, we restricted ourselves to patches of size $5\times 5$, $7\times 7$ and $9\times 9$.

The numerical tests were performed on a standard MacBook Pro (Retina, 13-inch, Early 2015), 2.9 GHz Intel Core i5, 8 GB 1867 MHz DDR3 using MATLAB 2016b.

### Discussion and outlook

We proposed in this section a combined algorithm to retrieve image contents from two images of illuminated manuscripts shown in Fig. 1 where very large regions have been damaged. At first, our algorithm computes an accurate segmentation of the inpainting domain which is performed by means of a semi-supervised method exploiting distinctive features in the image. Then, taking the segmentation result as an input, the procedure is followed by an exemplar-based inpainting strategy (upon suitable initialisation) by which the damaged regions are filled.

**Fig. 6:**
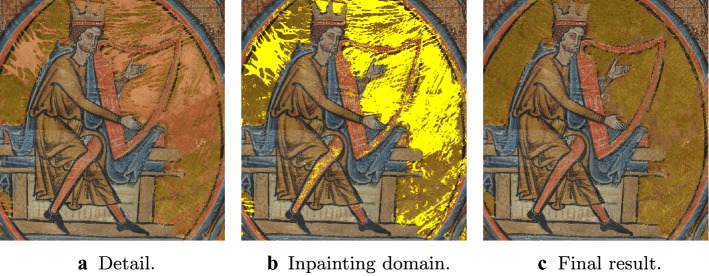
Inpainting of large image region with large damaged areas. Inpainting results of the combined model for a large detail ($1572\times 1681$ pixels) with large damaged areas. Patch size: $9\times 9$.

The results reported in Figs. 4 and 6 confirm the effectiveness of the combined method proposed. In particular, when looking at the difference between standard local (TV) image inpainting methods and the exemplar-based one we immediately appreciate the higher reconstruction quality in the damaged regions, especially in terms of texture information. The method has been validated on several image details extracted from the entire images, and has been shown effective also for very large image portions with highly damaged regions.

In term of computational times, the segmentations in Fig. 3 are obtained in approximatively 15 min. The inpainting results in Fig. 4 are obtained in about 3 min for patches of size $5 \times 5$ and about 7 min for patches of size $7 \times 7$. Overall the whole task of segmenting and inpainting the occluded regions takes approximatively 20 min per image of size $690 \times 690$. However, these results highly depend on the size of the image, the size of the inpainting domain and the size of the patches chosen.

Future work could address the use of different features for the segmentation of the inpainting domain with similar methodologies, such as for instance texture features [43]. Furthermore, at an inpainting level, we observe that the reconstruction of fine details in very large damaged regions (such as the strings of the harp in Fig. 6) is very challenging due to the lack of correspondence with similar training patches in the undamaged region. For solving this problem a combination of exemplar-based and local structure-preserving inpainting models could be used.

## Looking through the layers via osmosis filtering

**Fig. 7:**
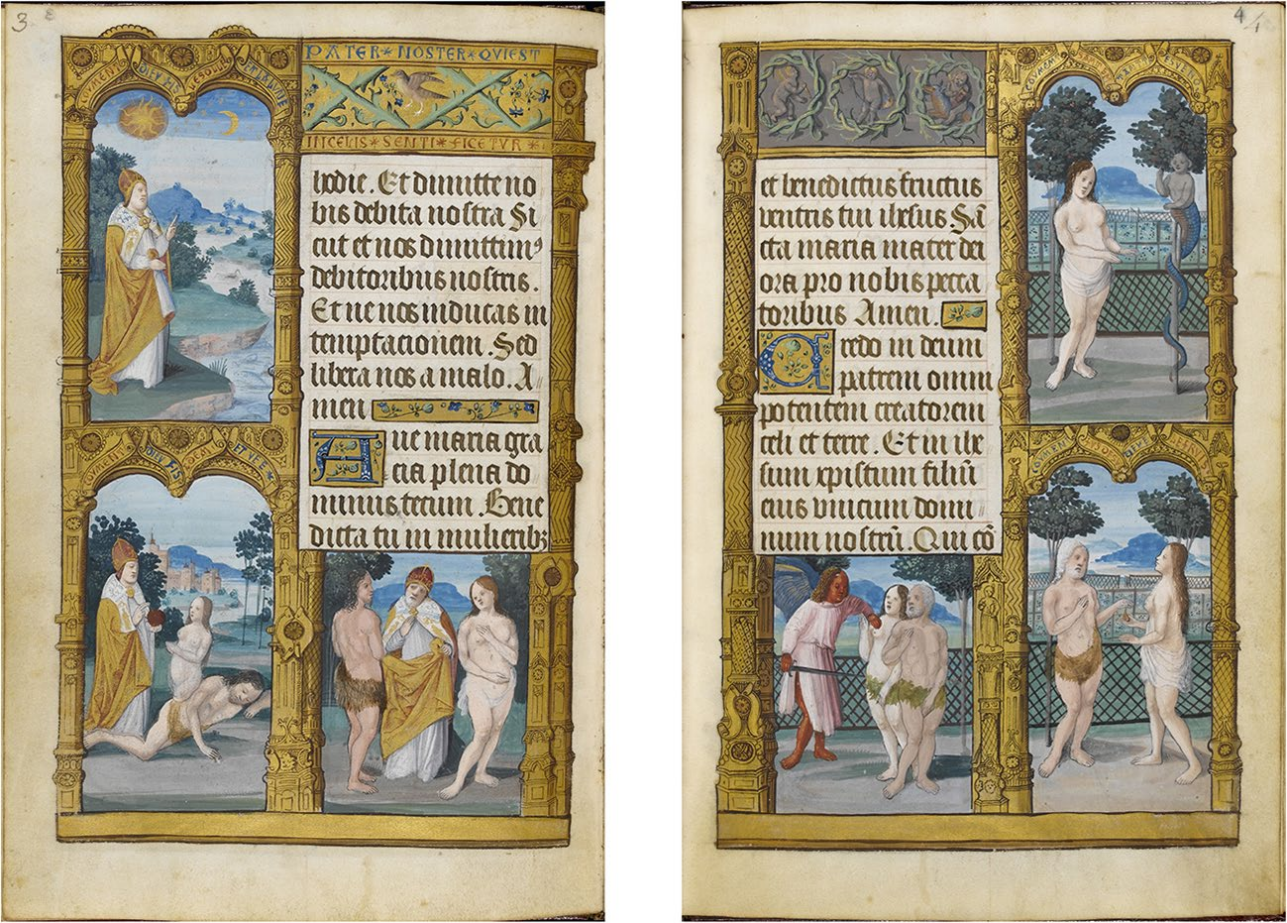
Illuminated manuscripts. Two illuminated pages from a manuscript touched up to cover Adam and Eve’s nudity, see “Looking through the layers via osmosis filtering” section for more details

**Fig. 8:**
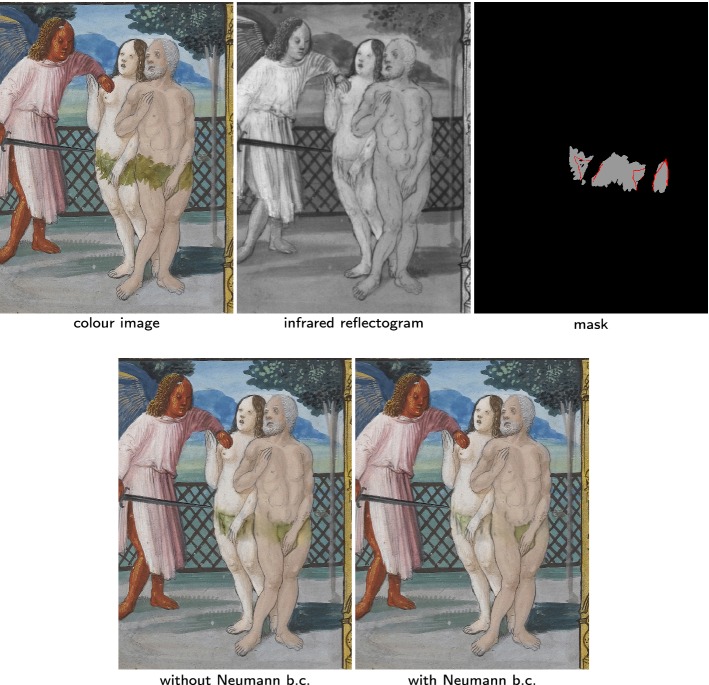
The over-paint is IR transparent (“IR transparent original pigments” section). We perform seamless cloning on the subdmain marked in gray on the mask. Bottom left: result without Neumann boundary conditions, the green from the fig leaf is diffused across the edges. Bottom right: cleaner result using Neumann boundary conditions. The Neumann boundary conditions (b.c.) are marked by red lines on the mask

In the previous section the image content in the damaged areas of the illuminations is completely lost and it was estimated only from the information available in the rest of the picture. This, however, is not the only kind of degradation encountered in the process of restoration of illuminated manuscripts. In some cases parts of an illumination are painted over. In this section we discuss as such an example the illuminations from the primer of Claude de France which illustrate the story of Adam and Eve in the garden of Eden. The two figures were originally depicted naked, as described in the book of Genesis but a later owner wanted them clothed with additional veils, leaves or beast skin added in the illumination, cf. Fig. 7. The use of infrared imaging as shown for instance in Fig. 8 allows to look through these added layers, unveiling hidden structural information underneath the painted layer. All the input colour images and their reflectogram are freely available on the Fitzwilliam museum website^5^ along with some more information about the manuscript, in particular the pigments used.

In this section we aim to fuse the details appearing in the near infrared reflectogram (IR) with the colours of the visible colour image, in particular the skin tones, to create a digital version of the illuminations as they could have looked before overpainting. Since we only have access to one near infrared reflectogram and we cannot chose the wavelength and have no information on the pigments used, we find ourselves in one of the following three situations: (i) the added cloth is transparent in the IR; (ii) the added cloth appears in the IR but without texture; (iii) the added cloth and its texture appear in the IR. The fact that the original pigments can also be IR transparent poses an additional challenge. For these different situations we use different methods all based on the use of the linear image osmosis model studied by Weickert et al. in [31].

In the following we first present the original parabolic linear osmosis equation studied in [31] and our slightly modified local elliptic formulation of osmosis [44]. Then we recall some of its common applications in image processing and finally apply our methods to digitally unveiling Adam and Eve in Claude De France’s Primer in each of the different situations (i)–(iii) described above (cf. “IR transparent original pigments”, “Over-paint with IR transparent texture” and “Non IR transparent over-paint texture: adding an inpainting step” sections).

### The Osmosis model

The osmosis model has been introduced in [31] as a non-symmetric generalization of diffusion filters and as a new tool for image processing problems such as seamless cloning and shadow removal. The original parabolic equation for this model is

9 $$\begin{aligned} {{u_t} = \Delta {u} - \text {div}({\varvec{d}}u)}. \end{aligned}$$

Here *u* is the solution we are looking for and $\mathbf {d}$ is a given vector field defined on the image domain $\Omega$ with values in ${\mathbb {R}}^{2}$ that we call the drift-field. Typically $\mathbf d$ encodes information from the gradient of the desired solution *u*, thus it serves as a guide to the diffusion process. For a given positive image *I*, when $\mathbf d = \mathbf {d}_I := \nabla I/I$, it turns out that *I* is a trivial steady state (i.e. a solution for $u_t=0$) of Eq. (9). Under this choice, the vector field $\mathbf {d}_I$ is called the *canonical* drift-field of *I*. Note that such drift-field is invariant to multiplicative changes of *I*.

Equation (9) is typically solved on the whole image domain under appropriate homogeneous Neumann boundary conditions. When applied to Cultural Heritage imaging this model has been successfully rendered computationally efficient by means of standard dimensional splitting techniques and applied, for instance, to Thermal-Quasi Reflectography (TQR) imaging and other similar applications in [45, 46].

In the following, we look directly for the steady state of the previous equation, i.e. the elliptic equation,

10 $$\begin{aligned} \Delta u = \text {div} (\varvec{d}u) \end{aligned}$$

and solve it on a small sub-domain *D* of the input image domain $\Omega$ with mixed boundary conditions as in [44]. Restricting ourselves to a small domain has two main advantages: first, most of the image is supposed to be left untouched; secondly, the computational cost is much smaller. Moreover, having mixed boundary conditions allows for more flexibility in adapting (10) to the problem at hand. In particular, Dirichlet boundary conditions enforce the colour values on $\partial D$ and a smooth transition of colour values across $\partial D$, which is appropriate if the image does not feature discontinuities (i.e. image edges) at the boundary of *D*. Neumann boundary conditions, on the other hand, prevent any diffusion across the boundary, ensuring clear colour discontinuities which is useful when the border of the mask is along an edge between two different colours appearing the same in the IR.

### Common applications of the model

The osmosis equation has been proposed for several tasks [31], the most common being shadow removal and seamless cloning as an alternative to Poisson editing [47]. All these tasks share the idea of manipulating the canonical drift-field $\mathbf {d}_I$ of one or more input images.

#### Shadow removal

The problem of shadow removal involves only one image and it is, as its name suggests, a process that takes as input an image with constant shadowed areas and gives as a result a shadow-free result. A constant shadow can be thought of as a multiplicative change in the domain of the shadowed region of the image. Since the canonical drift vector field is invariant to multiplicative change, the presence of the shadow is only encoded in the drift-field on the edge of the shadow. In an ideal case with a sharp shadow boundary, setting the drift field to zero there creates pure diffusion and results in a perfectly shadowless image [31].

#### Seamless cloning

Seamless cloning involves two input images that we will call the background image *g* and the foreground image *f*. This problem can be described as an improved copy-paste process where some information of *f* is copied in a sub-domain *D* of *g*. That is, one directly replaces in *D* the colour information of *g* by the colour information of *f*. This leads to a rough result where the boundaries of the pasted region are quite noticeable. Seamless cloning consists in doing this copy-paste process in such a way that the boundaries of the pasted region are no longer noticeable and the transition from *f* to *g* is smooth and natural. To this end we create a drift-field $\mathbf {d}$ from the canonical drift-fields $\mathbf {d}_{g}$ and $\mathbf {d}_{f}$ associated to *f* and *g*, respectively, so that:

$$\begin{aligned} \mathbf {d}(x) := {\left\{ \begin{array}{ll} \mathbf {d}_f(x)&{}\quad \text {if }\; x\in D,\\ \mathbf {d}_g(x)&{}\quad \text {if }\; x\in \Omega \setminus (D\cup \Omega _b),\\ \frac{\mathbf {d}_f(x)+\mathbf {d}_g(x)}{2}&{}\quad \text {if }\; x\in \Omega _b, \end{array}\right. } \end{aligned}$$

where $\Omega _b$ denotes the transition boundary. Once we have this composite drift-field, we can solve the osmosis equation on the whole image domain with Neumann boundary conditions [31] or, alternatively, only on the sub-domain *D* with Dirichlet boundary conditions [44]. On the one hand solving the equation on the whole image leads to the whole image being modified. On the other hand solving the equation only on *D* leaves the background image *g* untouched outside of *D*.

### Applications to illuminated manuscripts

In an ideal case, the added pigments do not appear on the IR while the colours to be restored are perfectly encoded in the IR. In this case the problem is reduced to a simple seamless cloning application with Dirichlet boundary conditions. The drift-field of the colour image is replaced by the one from the infrared image on the sub-domain to be restored. However, unfortunately, such an ideal case is uncommon. For the illuminations of the primer, we encounter rather different scenarios. For instance, when the added cloth is IR transparent or has no texture in the IR, the osmosis equation is enough to get a satisfying result. When the texture of the added cloth appears in the IR, the osmosis equation is no longer enough and we have to add an inpainting step to our method. We describe this in a greater detail in the following.

#### IR transparent original pigments

In Fig. 8, the IR along with a careful examination of the colour image reveals the existence of an original fig leaf under the added leaves of the over-paint. Here the over-paint is IR transparent so it should be a simple seamless cloning problem with the colour image being the background and the infrared being the foreground image. Yet, the colour distinction between the original fig leaves and the skin of Adam and Eve is hard in the IR. If we simply follow the seamless cloning method, we get back not only the skin colour but also the fig leaves colour from the small parts left untouched in the colour image. However because they appear the same in the IR, some diffusion occurs across the edges between the skin and the fig leaves. To prevent this, we enforce Neumann boundary conditions along these edges to prevent any such diffusion. The results with and without the use of Neumann boundary conditions (represented as red lines in the mask) are presented in Fig. 8.

#### Over-paint with IR transparent texture

**Fig. 9:**
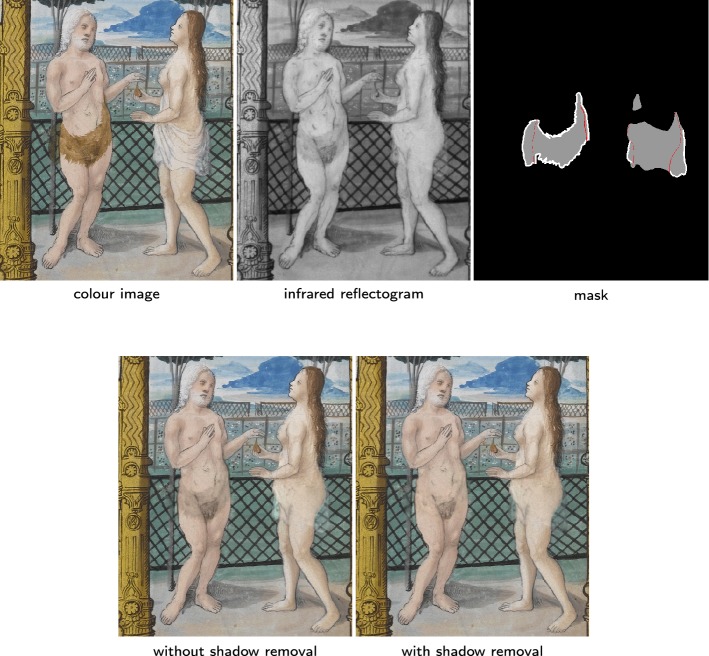
The texture of the over-paint is IR transparent (“Over-paint with IR transparent texture” section). Bottom left: we only applying the method of “IR transparent original pigments” section, the over-paint on Adam appears as some kind of shadow. Bottom right: after putting the drift-field to zero in the white areas of the mask, only some non IR transparent texture of the overpaint remains (on Adam’s hip and the part of Eve’s veil that covers the fence)

In Fig. 9, the added cloth on Adam is not IR transparent but it has little texture discernible on the IR and the original drawings appear clearly by transparency under it. This looks like a shadow in the IR as well as in the solution obtained with the method of the previous “IR transparent original pigments” section. Thus we mix seamless cloning with mixed boundary conditions and the shadow removal method. We replace the canonical drift-field of the colour image by the one of the IR in the region of interest. Then we put the drift-field to zero on the edge of the over-paint appearing in the IR. This method is illustrated in Fig. 9. The white lines of the mask are the areas where the drift-field is put to zero. In this figure we observe some transparent texture from the over-paint (over Adam’s hip and at the bottom of Eve’s veil). As expected, this texture appears in the final result.

#### Non IR transparent over-paint texture: adding an inpainting step

**Fig. 10:**
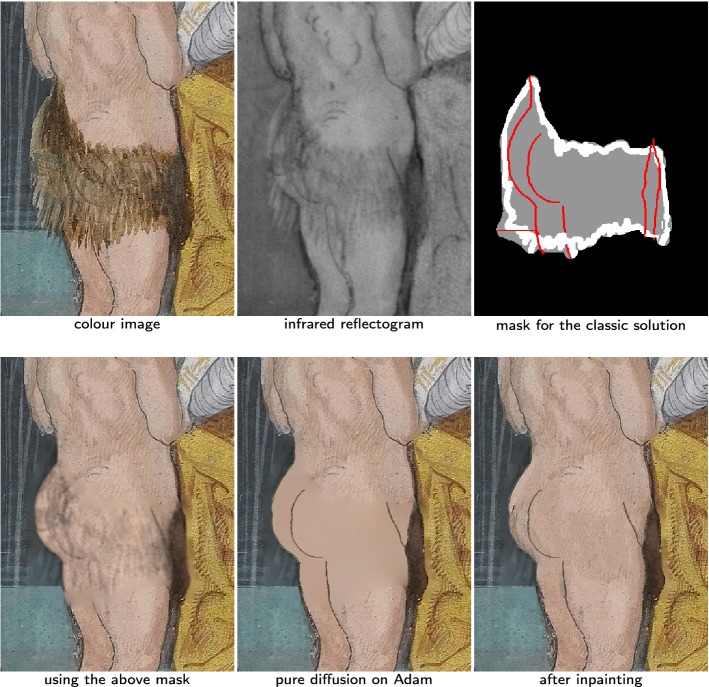
The texture of the over-paint appears clearly on the IR (“Non IR transparent over-paint texture: adding an inpainting step” section). Bottom left: using the method of “Over-paint with IR transparent texture” section, the texture of Adam’s skirt still appears clearly. Bottom center: we manually draw the underlying sketch and enforce pure diffusion on Adam’s skin. It leads to a complete loss of texture. Bottom right: after the inpainting step, the result looks more natural

In the case of Fig. 10, the IR adds some useful information to the colour image, as shown by the result obtained using the method from the previous “Over-paint with IR transparent texture” section but a large amount of the added skirt texture, visible in the IR, is also present. To get rid of this unwanted texture, we put the drift field to zeros on the area corresponding to Adam’s skin and manually segment the lines we want to keep. Note that this leads to a complete loss of texture in this region. To have a more natural looking result, we want to have some texture for the skin. While we can’t recover the original texture with our inputs, the untouched part of the illumination gives us some example of texture for Adam’s skin. This information is enough to use the exemplar-based inpainting algorithm described in “Exemplar-based inpainting” section, using as initialisation our result with missing texture. The final result on Adam’s skin has probably not much in common with the original painting but it appears natural enough, so it can help to get a better idea of the illumination in its original state.

### Preprocessing and parameters

As we just saw, such a complex restoration process necessitates significant user decisions. In fact the mask containing the sub-domain to be restored must be provided by the user as well as the edges along which Neumann boundary conditions should be applied and the sub-domain edges where the drift-field should be put to zero.

For our experiments we used the discretisation proposed in [31]. Then the linear system was solved using the MATLAB UMFPACK V5.4.0 LU solver. It took us at most 15 seconds to obtain the numerical solutions of the osmosis equation, our input images being respectively $901\times 1201$, $1001\times 1201$ and $952\times 1248$ for Figs. 8, 9 and 10. For Fig. 10 we only show a crop of our result of size $359\times 483$. For the inpainting step of Fig. 10, we used the implementation of the exemplar-based inpainting algorithm from [48]^6^ with the NL-medians method, $9\times 9$ patches, two scales and 4 iterations.

The numerical tests were performed on a standard MacBook Pro (Retina, 13-inch, 2017), 3,5 GHz Intel Core i7, 16 GB 2133 MHz LPDDR3 using MATLAB 2017b.

### Discussion and future work

We proposed in this section a method to digitally remove over-paint from an illumination using infrared information. Although we do not claim that our result perfectly corresponds to the original state of the illumination, we believe that nonetheless it offers an idea of its original state. For our applications the results are mostly satisfying, especially when the added pigments do not appear on the IR or when the addition doesn’t have too much texture visible in the IR. As the process necessitates some important user decisions, it is preferable to have input from an expert. From the IR alone we can only make educated guesses. Only outside information from an expert allows us to know which pigments have been over-painted, from examination under a microscope for example. This method is fast enough to allow fine tuning by the user as depending on the result the mask can be repeatedly improved. The quality of the output is highly dependent on the infrared wavelength and the pigments used for both the original painting and the over-paint.

Future work should address these difficulties and test the method on a larger dataset. An easy improvement would be to have an IR with the same resolution as the colour image to prevent the blur effect that we can observe. For the mask creation phase, a more automated segmentation detection could be inserted to have a first guess. In this work, we have only used the visible image and a single IR. Better results may be obtained by using several IR’s where the wavelengths are chosen depending on the pigments used. In such a situation, the expert would only have to specify for each area which IR should be used.

## Creating a 3D virtual scene from illuminated manuscripts

In recent years, certain museums and companies have taken a step beyond using digital technology to *restore* historic artwork, and have instead created 3D or animated versions of historic artwork that can *only* be experienced digitally. For example, the British Museum’s Hutong Gallery recently created a 3D version of the 1623 painting “Reading in the Autumn Mountains” (originally painted during the Ming dynasty by the artist Xiang Shengmo). A video in which the viewer flies through the 3D painting can be found on their website [49]. Another example, which was shown at the Taipei Flora Expo in 2010/2011, features a Song Dynasty painting that was converted into an animation [50, 51]. In this case, the animated painting was displayed on a specially designed screen, twenty feet wide and more than 360 feet long, mounted on the wall of the exhibition center. Finally, the Shanghai based company Motion Magic has created 3D versions of the paintings of Vincent Van Gogh, which viewers can walk around inside after putting on virtual reality goggles [52, 53]. The result of these efforts is both a new kind of art and a new way of interacting with art. This trend is likely to get stronger as virtual reality becomes more mainstream and the demand for VR content increases.

**Fig. 11:**
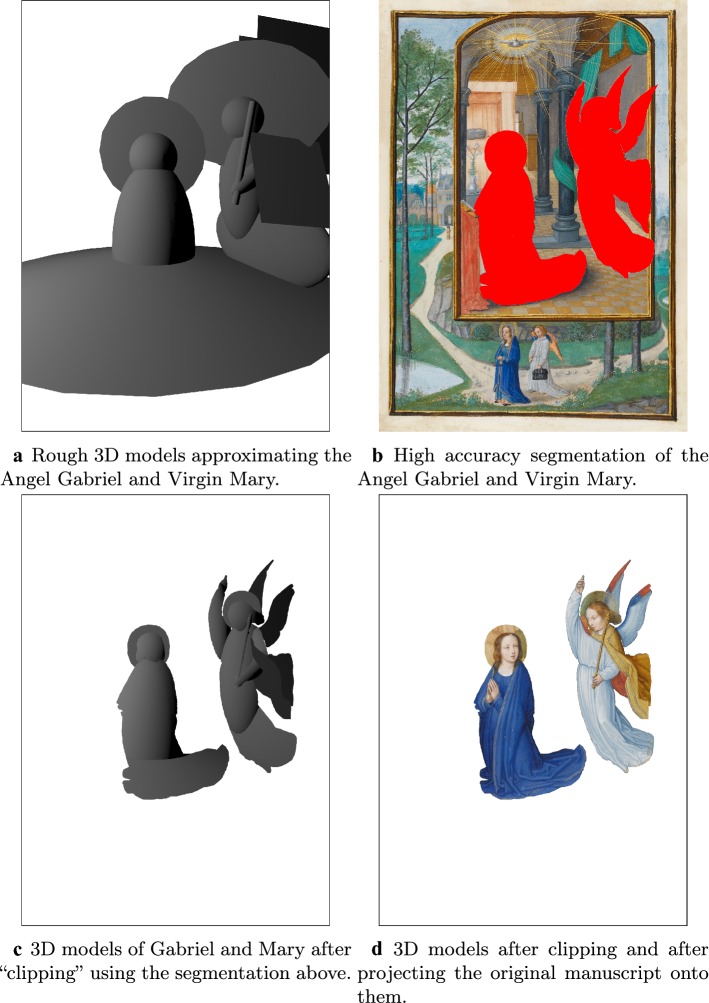
3D conversion Pipeline. Here we illustrate steps one to three of the 3D conversion pipeline presented in the “Overview of a 3D conversion pipeline” section. First, in **a**, rough 3D geometry is generated for all objects in the scene (here, only the Virgin Mary and Angel Gabriel are shown). Next, in **b**, accurate masks are generated for all objects (again, only Mary and Gabriel are shown). In **c**, the camera is turned into a projector and the masks from **b** onto the rough 3D geometry from **a**. This projection is then used to “clip” the 3D models by throwing away the portion of the geometry not falling within the projection. Finally, in **d**, the clipped geometry is “painted” by projecting the original image onto it

**Fig. 12:**
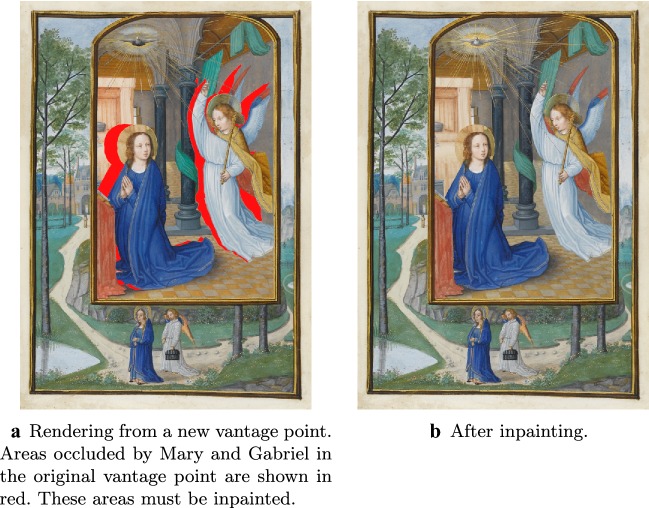
3D conversion Pipeline continued. Here we illustrate steps four and five of the 3D conversion pipeline presented in "Overview of a 3D conversion pipeline". In **a**, we have rendered the 3D scene from a new vantage point. This will be the left eye view of a stereo pair in which the right eye view is the original manuscript. Areas in red are occluded by Mary and Gabriel in the original manuscript and must be inpainted. In **b**, we see the result of inpainting, which in this case is done using a combination of Content-Aware Fill and manual copy pasting of image patches.

**Fig. 13:**
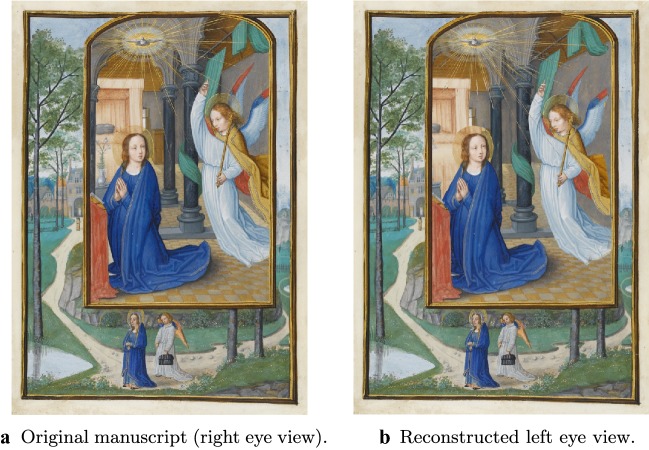
3D conversion of an illuminated manuscript. The illuminated manuscript considered here is *Annunciation* by Simon Bening, Fitzwilliam Museum, MS 294b, Flanders, Bruges, (1522–1523). The restored manuscript (a) is converted into a stereo 3D pair. To view the resulting stereo 3D image without glasses, first cross your eyes so that each image splits in two. Make the middle two images overlap, and then bring the superimposed image into focus (try varying your distance from the computer screen)

**Fig. 14:**
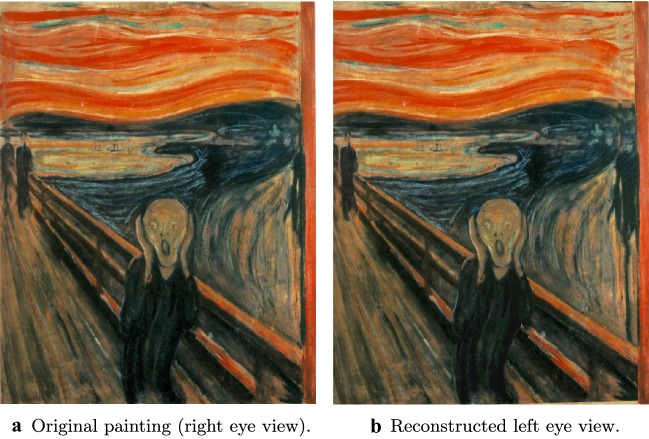
3D conversion of * The Scream*. The original painting (a) is converted into a stereo 3D pair. To view the resulting stereo 3D image without glasses, first cross your eyes so that each image splits in two. Make the middle two images overlap, and then bring the superimposed image into focus (try varying your distance from the computer screen)

In this section, we demonstrate the potential of these approaches by converting an illumination from the manuscript *Annunciation* by Simon Bening, Fitzwilliam Museum, MS 294b, Flanders, Bruges, (1522-1523), as well as the painting *The Scream* by Edvard Munch into stereo 3D (see Figs. 13 and 14). We do so using a 3D conversion pipeline originally developed for the conversion of Hollywood films. There, one is given the video shot from camera position $p \in \mathbb {R}^3$ and orientation $O \in SO(3)$ (corresponding to, for example, the left eye view), and the objective is to generate a plausible reconstruction of the video as it would appear from a perturbed position and orientation $p+\delta p \in \mathbb {R}^3$, $O+\delta O \in SO(3)$ (corresponding to the view from the other eye). In some cases *p* and *O*, along with other relevant camera parameters such as field of view, may be given. In other cases, they must be estimated. In our case the process is the same, except that we have a manuscript (or painting) rather than a video. However, this introduces a subtle difference. In the case of converting a video shot with a real camera, although we might not know the associated camera parameters, we at least knew that they *exist*—but here, because the input is drawn by a human, existence is not given. In particular, depending on the artist, the drawing may or may not obey the laws of perspective. This is particularly noticeable in the case of *The Scream*—see Fig. 16.

### Overview of a 3D conversion pipeline

**Fig. 15:**
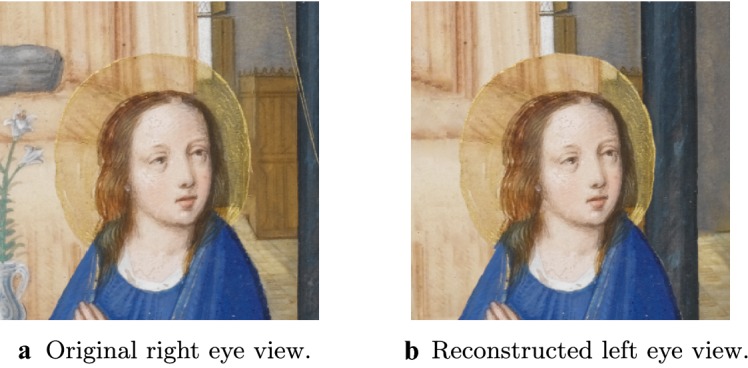
3D conversion pipeline failure when applied to semi-transparent surfaces. Closeup of the halo of the virgin Mary in the original right eye view (**a**) and the reconstructed left eye view (**b**). In **a**, we are able to see part of the background—in this case Mary’s bed—through her halo. In **b**, the same chunk of background is incorrectly carried over to the new location, obscuring the actual background

Here we briefly go over the 3D conversion pipeline used in this paper. The steps of the pipeline are illustrated in Figs. 11 and 12. For more details, please see [33] or [28, Ch. 9.4].

1. Generate a rough but plausible 3D model of the scene, including a virtual camera with plausible parameters (parameters include position, orientation, field of view, possibly lens distortion, etc) placed within it. The 3D models do not have to be perfect, and are typically made a little larger than the objects they correspond to. This is because they will be “clipped” in step three. See Fig. 11a, where we show rough 3D models used for the Virgin Mary and Angel Gabriel.
2. Generate accurate masks for all objects in the scene. This is typically done by hand, but could also be done with the help of segmentation algorithms that are then touched up. See Fig. 11b, where we show masks for the Virgin Mary and Angel Gabriel.
3. The camera is then transformed into a projector, which is used for two purposes. Firstly, the masks from the previous step are projected onto the rough 3D geometry from step 1, and used—much like a cookie cutter—to “clip” the geometry, throwing away the portion that is unneeded. See Fig. 11c, where we illustrate this for the 3D models of Mary and Gabriel. Secondly, the original image is then used as texture by projecting it onto the clipped geometry, as in Fig. 11d.
4. One or more new virtual cameras are added to the scene. If the original camera is taken to be either the right or left eye, then one additional virtual camera corresponding to the other eye is needed. However, sometimes the original camera position is taken to be half way between the two eyes, so that two virtual cameras (corresponding to the left and right eyes) are needed. These camera(s) will be used to render the 3D scene from one or more new viewpoints, in order to create a stereo pair.
5. Because the new camera(s) will typically see bits of background previously hidden behind foreground objects in the original view, inpainting of occluded areas is required. This is typically done using a toolbox of inpainting algorithms that are then touched up by hand. In our example, inpainting was done in Photoshop, using a combination of Content Aware fill and manual copy-pasting of patches by hand. See Fig. 12a, b, where we show the rendering of *Annunciation* from a new view, including in (a) the areas originally occluded by Mary and Gabriel, and in (b) the result after inpainting these areas. In reality, as this scene contains many more 3D objects than just Mary and Gabriel, what is shown in 12a is just a sampling of the inpainting problems that need to be solved.

Steps one, two, and the first half of step three can be thought of as generating a *depth map* for the image. The rough geometry generated in step one provides the smooth component of the depth map, while the masks generated in step two define the depth discontinuities, which are imposed on the geometry by the “clipping” in step three. Because the human eye is most sensitive to depth discontinuities, these have to be very accurate, but the 3D models do not. For example, in the conversion of Fig. 13a, the virgin Mary is modelled using just a few simple geometric primitives including an ellipsoid for her body, a sphere for her head, a cylindrical halo and a cone for the bottom of her dress. This is illustrated in Fig. 11a, where the geometry of the Angel Gabriel (also consisting of simple geometric primitives) is also shown.

### Results and future work

The results of our 3D conversion of *Annunciation* are presented in Fig. 13, where we show the original manuscript (assumed to be the right eye view) side by side with the reconstructed left eye view. Similarly, Fig. 14 shows our results for the 3D conversion of *The Scream*. Please see the video files provided in Additional files 1 and 2 atfor animated versions of our results. The conversion of *Annunciation* illustrates a limitation of the pipeline we have used: it does not handle partially transparent objects properly. In this case, bits of background in the original right eye view are visible through the halos of both the virgin Mary and the angel Gabriel. In particular, in the original right eye view, a bit of Mary’s bed is visible through her halo. When rendered from the new left eye vantage point, we should now be seeing the window through her halo, but instead we continue to see the bed. See Fig. 15 for a closeup of this defect. To overcome this, one could modify the pipeline in the “Overview of a 3D conversion pipeline” section to first decompose semi-transparent objects into two images (in this case, the pure halo and the background). This is something we would like to investigate in the future.

**Fig. 16:**
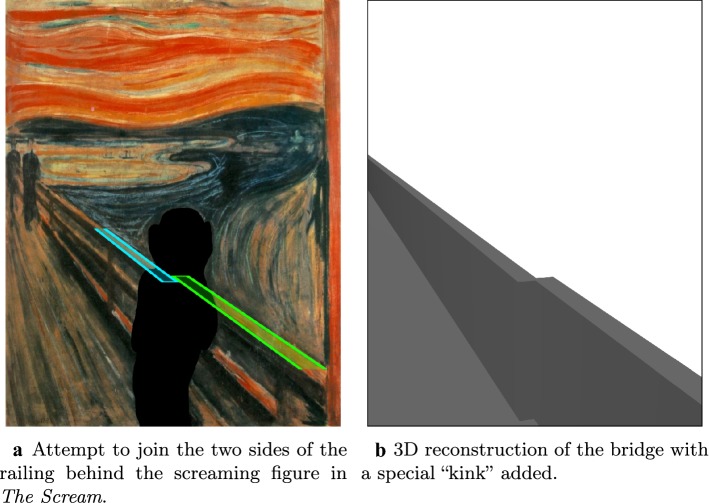
Perspective failure in *The Scream*. In the process of coverting *The Scream* into 3D we discovered, as in **a**, that the railing of the bridge in the painting does not obey the laws of perspective. To get around this issue, we had to introduce a “kink” into our 3D model of the bridge, as in **b**

The conversion of *The Scream* illustrates a nuance arising in the 3D conversion of paintings—namely that paintings may not obey the laws of perspective. In this case, due to the failure of perspective, it is not possible to extrapolate the railing of the bridge into the occluded area behind the screaming figure without introducing a bend or “kink”. This is illustrated in Fig. 16 where we also show the “kink” we had to introduce into the 3D model of the bridge in order to make 3D conversion of this painting possible.

## Conclusion

An adequate mathematical analysis and processing of images arising in the arts and humanities needs to meet special requirements:

- There is often particular domain expertise which any analysis should ideally make use of. For instance, when digitally restoring an image, the integration of related images such as paintings from the same artist, could be taken into account. In what we have discussed this concept is used to the extent that a dictionary of characteristic structures in the undamaged part of the illuminations was created and used to fill in the lost contents in the damaged regions, compare Figs. 4, 6. This could be driven much further, expanding the dictionary by illuminations or details of illuminations from the same artist.
- The results achieved in Figs. 10, 9, 8 show a possible use-case for scientific imaging in art restoration or art interpretation. Indeed, we believe that the integration of different types of scientific imaging such as infrared imaging, are likely to give benefit to image analysis methods and so the latter should be able to capture those.
- Explainability of results is crucial. There is clearly a balancing act to be made between hand-crafted analysis that captures expert knowledge and a black-box, data-driven image analysis approach. In particular, the latter should ideally have an interpretable mathematical representation that gives rise to new conclusions. In this paper we have solely considered model-based and hence explainable solutions to art restoration and interpretation problems. The growing emergence of deep learning solutions to various image analysis tasks provides an alternative approach to these problems, at the moment however without a proper explanation.
- Relevant characteristics are often hidden in very fine details of the artwork, like a brushstroke in a painting. Capturing these fine details in a digital format results in high-resolution images that an image analysis method should be capable of processing. This means there is a demand for computationally-efficient image analysis methods.
- Digital processing and manipulation of artwork opens up a myriad of possibilities of analysing and processing, but also of experiencing, understanding and reinterpreting artwork. As an example we have shown 3D conversion and its possible use-cases in the presentation of art, cf. Fig. 13 for instance.

With the above in mind, we have discussed a selected subset of mathematical approaches and their possible use-cases in the restoration and interpretation of illuminated manuscripts. These approaches are not perfect yet by all means and there is plenty of room for improvement, compare our discussion in “Discussion and outlook”, “Discussion and future work” and “Results and future work” sections.

## Additional file

**Additional file 1.** 3D Conversion of* Simon Benning, Annunciation*: Here we visualize a 3D version of Simon Benning,Annunciation with an animation that loops between the left and right eye viewpoints.

**Additional file 2.** 3D Conversion of Edvard Munch's *The Scream*: Here we visualize a 3D version of Edvard Munch's *TheScream* with an animation that loops between the left and right eye viewpoints.

## Footnotes

1 For our computation we used the inbuilt MATLAB Chan-Vese segmentation code.

2 The popular *k*-means algorithm is classically used to partition a set of *N* observations (in our case, the image pixels) into *K* clusters such that each observation is grouped to the cluster having the closest mean value. Each resulting cluster is labelled in order to distinguish it from the others.

3 The code is freely available at Image Processing On-Line (IPOL) Journal: https://doi.org/10.5201/ipol.2017.189.

4 The code is freely available at Image Processing On-Line (IPOL) Journal: https://doi.org/10.5201/ipol.2012.g-tvi

5 http://www.fitzmuseum.cam.ac.uk/illuminated/manuscript/discover.

6 The code is freely available at IPOL: https://doi.org/10.5201/ipol.2015.136
